# Correction: Coexistence of relaxor ferroelectricity and magnetism in hexagonal high-entropy manganite thin films

**DOI:** 10.1039/d6tc90146h

**Published:** 2026-10-30

**Authors:** Duncan Miertschin, Sundar Kunwar, Balaram Regmi, Poshan Kandel, Peter Bencok, Zheng Gai, Luis M. Martinez, Prashant Padmanabhan, Lide Yao, Yu-Wei Chen, Yen-Lin Huang, Sebastiaan van Dijken, Alessandro R. Mazza, Nick A. Shepelin, Alan Farhan

**Affiliations:** a Department of Physics and Astronomy, Baylor University, One Bear Place TX 76798 Waco USA alan_farhan@baylor.edu; b Center for Integrated Nanotechnologies, Los Alamos National Laboratory Los Alamos New Mexico 87545 USA; c Diamond Light Source Didcot Oxfordshire OX11 0DE UK; d Center for Nanophase Materials Sciences, Oak Ridge National Laboratory Oak Ridge Tennessee 37831 USA; e OtaNano-Nanomicroscopy Center, Aalto University School of Science P.O. Box 15100 FI-00076 Aalto Finland; f Department of Materials Science and Engineering, National Yang Ming Chiao Tung University Hsinchu 300093 Taiwan; g Department of Applied Physics, Aalto University School of Science P.O. Box 15100 FI-00076 Aalto Finland; h Materials Science and Technology Division, Los Alamos National Laboratory Los Alamos NM 87545 USA; i PSI Center for Neutron and Muon Sciences, Paul Scherrer Institute Forschungsstrasse 111 5232 Villigen PSI Switzerland

## Abstract

Correction for ‘Coexistence of relaxor ferroelectricity and magnetism in hexagonal high-entropy manganite thin films’ by Duncan Miertschin *et al.*, *J. Mater. Chem. C*, 2026, https://doi.org/10.1039/d6tc01787h.

The authors regret that in the original article, the Burns temperature was given as *T*_B_ = 420 ± 20 K in the caption of Fig. 6 and the sentence beginning ‘Deviations from Curie–Weiss behavior occur …’ on Page 8 of the PDF proof. It was not made clear that this value was an upper limit and that it ranges from 360 K to 420 K, depending on frequency.

The Royal Society of Chemistry apologises for these errors and any consequent inconvenience to authors and readers.

